# Host-Informed Engineering of Glycosylation in Komagataella phaffii for Human Biantennary N-Linked Glycans

**DOI:** 10.21203/rs.3.rs-10329002/v1

**Published:** 2026-07-20

**Authors:** Yuchen Yang, Christopher A. Naranjo, Neil C. Dalvie, Joshua A. Hinckley, Shuting Shi, J. Christopher Love

**Affiliations:** Massachusetts Institute of Technology; Koch Institute for Integrative Cancer Research At MIT; Massachusetts Institute of Technology; Koch Institute for Integrative Cancer Research At MIT; Massachusetts Institute of Technology; Massachusetts Institute of Technology

## Abstract

*Komagataella phaffii* is a common alternative host to mammalian cell lines for heterologous production of recombinant proteins due to its cost-effectiveness and accelerated development. Its native N-linked glycosylation, however, yields high-mannose structures that differ from those commonly found on secreted recombinant proteins used in biopharmaceuticals. The feasibility of humanizing *K. phaffii*’s glycosylation pathway has been demonstrated, but routine engineering of this attribute remains underdeveloped. In this study, we reconstructed a humanized glycosylation pathway capable of producing the biantennary GlcNAc2Man3GlcNAc2 (G0) glycan structure. We report a previously undescribed synthetic lethality related to the overexpression of *C. elegans* α1–2 mannosidase (MNS1) and the deletion of the native OCH1 gene that can be mitigated by using weaker native promoters for genes modified in the glycosylation pathway. Engineered strains exhibited reduced growth rates compared to unmodified strains, and RNA sequencing revealed upregulated stress response and cell cycle pathways in glycoengineered strains. Through transcriptomics-guided gene knockouts, particularly in the MAPK signaling cascade, we partially restored growth in a G0-engineered strain. This study demonstrates a targeted, host biology-informed strategy for improving glycoengineering in *K. phaffii* by combining a CRISPR-Cas9 genome-editing system and transcriptomic analysis.

## Introduction

The methylotrophic yeast *Komagataella phaffii* is widely used for production of heterologous proteins in both academia and industry. The increasing demand for global accessibility [1], sustainable manufacturing [2], and accelerated development [3] has further spurred interest in alternative hosts like *K. phaffii* for the production of high-value pharmaceutical proteins [4]. Mammalian cells such as Chinese Hamster Ovary (CHO) cells, however, are the most commonly used host for manufacturing today for certain proteins used in biopharmaceuticals and vaccines because these cells yield profiles of N-linked glycans that are similar to those from humans and needed for folding, stability, and efficacy [5], [6]. In contrast, yeast like *K. phaffii* natively produce high-mannose glycans that may confer unwanted *in vivo* immunogenicity, resulting in the target molecule’s fast clearance and poor efficacy [7]. Deliberate engineering to remodel the pathways for glycosylation offers an alternative approach to address this shortcoming [8].

Humanization of *K. phaffii* glycosylation, especially its N-linked glycosylation pathway, involves the inactivation of the native glycosylation pathway and the integration of heterologous glycosylation genes [9]. This engineering process, however, has been notably difficult. Combinatorial libraries of subcellular localization sequences and catalytic domains have been screened to select the most effective heterologous glycosylation genes, often driven by strong constitutive promoters like GAPDH promoter to maximize their expression [10]. Additionally, glycoengineered strains suffer fitness defects, including slower growth [11], decreased stationary phase cell density [12], and increased propensity for lysis [13]. Such disadvantages in cell growth and cultivation robustness limit the application of this yeast. Research into improving the performance of glycoengineered strains has been limited and has often required extensive clone-screening: UV-directed random mutagenesis has yielded mutants with increased growth [13]; truncated variants of heterologous genes, especially *MNS1* (the α1–2 mannosidase responsible for the initiation step in the humanized glycosylation pathway) has resulted in enhanced viability of glycoengineered strains [14].

With new gene-editing technologies like CRISPR-Cas9 and advanced -omics techniques like whole transcriptome sequencing, we hypothesized that a targeted and host-informed approach to glycoengineering could improve humanization of glycosylation pathway for *K. phaffii*. Here, we have reconstructed the humanized glycosylation pathway to produce GlcNAc2Man3GlcNAc2 (G0) glycans on recombinant proteins. We also found a previously unreported synthetic lethality: The overexpression of *C. elegans* α1–2 mannosidase *MNS1* is incompatible with the deletion of native α1–6 mannosyltransferase *OCH1*. We show this lethality could be addressed by expressing the wild-type *MNS1* and the other genes in the humanized pathway with weaker, native biology-informed promoters to achieve glycan homogeneity without further negative effects on growth. RNA sequencing confirmed that wildtype *K. phaffii* was more metabolically active, whereas cell cycle regulation and stress response-related signaling pathways were upregulated in the glycoengineered strain. Guided by gene set enrichment analysis with transcriptomic data, we partially restored growth defects in a glycoengineered strain through gene knockouts within the yeast MAPK cascade pathway.

## Materials and Methods

### Yeast vectors and strains

All strains were derived from wild-type *Komagataella phaffii* (NRRL Y-11430). The DNA fragment containing 6His-tagged K3 peptide was codon optimized, synthesized (Integrated DNA Technologies), and cloned into a custom vector with the methanol-inducible promoter P_AOX1_ for roll-in integration [15]. *K. phaffii* strains were transformed as described previously [16].

### Glycosylation pathway engineering

Genetic manipulation via CRISPR-Cas9 system was done as previously described.[16] Guide RNAs were designed targeting their respective integration sites using the ATUM gRNA Designer tool (www.atum.bio), and the gRNA sequence of the CRISPR-Cas9 plasmid was substituted via site-directed mutagenesis (NEB).

Integration cassettes containing *MNS1*, *YEA4*, *GNT1*, *MNS2*, and *GNT2* were codon optimized, synthesized (Integrated DNA Technologies), and cloned into a custom vector with appropriate promoters. They were targeted to intragenic regions of the genome adjacent to genes *PFK1*, *GQ67_04576*, *GQ67_03878*, *GQ67_04704*, and *GQ67_05172*, respectively, using 500 bp flanking sequences for homologous recombination. For *K. phaffii* native promoters, the promoter region was defined as the sequence 1kb upstream of the corresponding gene or till the opening reading frame of the adjacent gene, whichever is shorter. Modification of the vector, including the replacement of promoter and/or integration site, was performed using PCR and Gibson assembly (New England Biolabs).

Cells were transformed via co-electroporation of 250ng of CRISPR-Cas9 plasmid and 2.5μg of linear integration cassette (generated through PCR amplification and column purification). For each transformation, eight to sixteen colonies (or more if appropriate) were randomly selected for genomic DNA extraction. The integrations were verified by amplifying the targeted loci from each genomic DNA sample, and Sanger sequencing (Genewiz) was performed for sequence confirmation.

### Yeast cultivations

Strains were grown in 3mL cultures in 24-well deep well plates (ambient temperature, 600 rpm). Cells were cultivated in BMGY (buffered glycerol complex media, consisted of potassium phosphate buffer pH 6.5, 1.34% nitrogen base without amino acids, 1% yeast extract, 2% peptone). Cells were inoculated at 0.1 OD600, outgrown for 24 hours with 4% (v/v) glycerol, centrifuged to pellet, and then resuspended in fresh media with 3% (v/v) methanol to induce recombinant gene expression. Supernatant samples were collected after 24 hours of production, filtered, and analyzed. For glycoengineered strains with *Δoch1* genotype, both biomass accumulation and protein production were extended to 48 hours.

### Protein purification and glycan characterization

The filtered supernatant samples were diluted 1:1 with Ni-IMAC (nickel-immobilized metal affinity chromatography) binding buffer (25mM imidazole, 25mM sodium phosphate, 500mM sodium chloride, pH 7.4) and purified on GE-AKTA pure system with 1-mL His-tag protein purification column (HisTrap HP, Cytiva). After sample loading, the column was equilibrated with and washed with the binding buffer before eluting with elution buffer (500mM imidazole, 25mM sodium phosphate, 500mM sodium chloride, pH 7.4).

Intact protein LC-MS was performed as described previously (cit.). Mass spectra were processed using MassHunter Bioconfirm software (Agilent Technologies) with a deconvolution range of 10–15 kDa, using a mass step of 0.5 Dalton.

### Growth rate assay

Strains were grown in shake flasks in YPD (BD Difco) at 30°C and 250rpm (overnight for wildtype, and over two nights for glycoengineered strains). They were then inoculated in 100μL BMGY supplemented with 4% (v/v) glycerol to an initial OD of 0.01 (wildtype) or 0.05 (glycoengineered) strains in a Nunclon^®^ Delta surface 96-well plate. Cultures were subsequently grown in a microplate reader (Tecan) with shaking at 1000rpm and ambient temperature for 24 hours, with OD600 measurements taken every 30 minutes. Growth data during exponential phase was fit to an exponential function for growth rate calculation.

### Transcriptome analysis

Cells were inoculated at 0.1 OD600 at 3mL plate scale and harvested after 18 hours of biomass accumulation in BMGY supplemented with 4% (v/v) glycerol. RNA was extracted and purified according to the Qiagen RNeasy 96 kit. RNA quality was analyzed on Agilent BioAnalyzer to ensure RNA quality number > 6.5. RNA was reverse transcribed with Superscript III (ThermoFisher) and amplified with KAPA HiFi HotStart ReadyMix (Roche). RNA libraries were prepared using Nextera XT DNA Library Preparation Kit with the Illumina DNA/RNA UD Indexes Set A. They were then sequenced on Illumina Nextseq to generate paired reads of 50bp (read 1) and 50bp (read 2). Sequenced mRNA transcripts were demultiplexed using sample barcodes, aligned to the wildtype *K. phaffii* genome (strain Y-11430) and exogenous transgenes, and quantified using Salmon version 1.6.0 [17]. Gene level summaries were prepared using tximport version 1.24.0 [18] running under R version 4.2.1. Pathway enrichment analysis was carried out as previously described [19].

### Alcian blue, Congo red, and Calcofluor white assays

Alcian blue assay was carried out as described previously [20] with minor modifications. Briefly, strains were growth in YPD (overnight for wildtype, and over two nights for glycoengineered strains) at 30°C and 250 rpm. 1 OD600 equivalent of cells were then pelleted and washed once with and resuspended in 100μL 0.02N HCl. The resuspensions were transferred into a 96-well V-bottomed PCR plate (Eppendorf), and 100μL of Alcian blue solution was added. After incubation at room temperature for 15 minutes, the plate was centrifuged at 3,100 g for 15 minutes.

Congo red and Calcofluor white assays were carried out similarly [20] but with final concentrations of Congo red and Calcofluor white at 25 mg/L and 15 mg/L, respectively.

#### MAPK signaling pathway knockout

Essentiality score was based on a previously described dataset [21] which used a CRISPR-Cas9 genome-wide knockout library to investigate gRNA drop-out after transformation. Twenty genes in the signaling pathway with the highest normalized enrichment score were chosen, and 0.75 non-essentiality score was selected to be the cut-off, generating eight genes for knockout target. Non-essentiality score was defined as the adjusted *p*-value of gRNA drop-out, so that closer the *p*-value is to 1, the less essential the gene is. Gene knockouts were carried out as previously described [22].

## Results

In reported glycoengineering of *K. phaffii*, many genes for humanized glycosylation were constructed using the cDNA libraries of the host organisms and expressed under the control of strong constitutive or methanol-inducible promoters such as *GAPDH* promoters, likely to maximize the expression of the heterologous glycosylation pathway [10], [23], [24]. As different organisms have different codon usage preference, the native sequences of glycosylation enzymes from higher eukaryotic organisms (including *Homo sapiens*, *Rattus norvegicus*, and more) can contain codons rarely used in *K. phaffii*. Codon optimization and gene synthesis technologies, however, have improved in recent years and can in turn improve recombinant protein and heterologous pathway expression [25]. Furthermore, existing engineering approaches often depend on the repeated recycling of multiple auxotrophic or antibiotic markers [23], [26], which leaves genomic scars and potentially introduces additional metabolic stress. To circumvent this problem, we previously adapted and optimized the CRISPR-Cas9 system for *K. phaffii*, allowing for fast and markerless genome editing [27], [28].

To engineer the glycosylation of the *K. phaffii*, we codon-optimized and integrated the first three genes in the humanized glycosylation pathway, α1–2 mannosidase *MNS1*, UDP-GlcNAc transporter *YEA4*, and UDP-GlcNAc transferase *GNT1*, based on their respective previously reported best-performing constructs [10]. Strong constitutive promoters P_GAPDH_, P_TEF1_, and P_ENO1_ were used to drive their expression. Finally, we disrupted the native alpha-1,6-mannosyltransferase *OCH1* (the gene responsible for the initiation of *K. phaffii* native outer chain hypermannosylation). This deletion strongly reduces the growth rate of the strains, and our approach here minimized the impact of this change compared to previous glycoengineering strategies which started with this knock-out [27].

**Figure 1:**
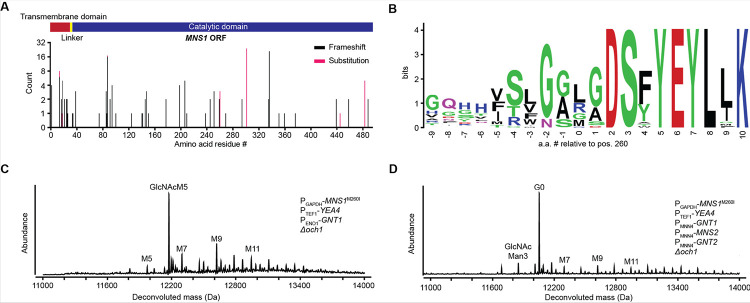
Discovery and resolution of a synthetic lethality during glycosylation pathway construction. A) Number of spontaneous mutations in the *MNS1*open reading frame after *OCH1* knockout, as confirmed via Sanger sequencing. B) Logo plot of amino acid residues surrounding the methionine of interest (M260 in the integrated transgene) across different organisms. C) Mass spectrum of K3 peptide secreted from a GlcNAcMan5-glycoengineered strain. D) Mass spectrum of K3 peptide secreted from a G0-glycoengineered strain.

To benchmark the resulting glycoengineered strain, we expressed K3 peptide—a highly soluble peptide with a single N-linked glycosylation site derived from human plasminogen Kringle 3 domain. In strains with disrupted *OCH1*, however, we did not detect the presence of GlcNAcMan5 glycan on secreted K3 via intact protein LC-MS (**Figure S1**). Since the glycan profile mainly comprised of high mannose structures ranging from Man8 to Man12, we suspected that the heterologous *MNS1*, responsible for cleaving Man8 to Man5, lost its enzymatic activity. By Sanger sequencing the transformants’ *MNS1* integration, we discovered spontaneous mutations in its coding sequence. We further confirmed that no *MNS1* mutations existed in the parent strain (with intact *OCH1*) and that strains with additional integration of *MNS2* and *GNT2* also yielded mutations in *MNS1* ORF upon *OCH1* knockout. Combining all *OCH1* knockouts across different glycoengineered strains, we screened a total of 158 colonies, 121 of which had frameshift mutations in *MNS1*, and the remaining 37 had amino acid substitutions in the gene. These mutations occurred throughout *MNS1* ORF at 47 unique amino acid residues (Fig. 1A). The spontaneous occurrence of unlinked mutations in the *MNS1* coding sequence suggested an incompatibility between the disruption of *OCH1* and overexpression of *MNS1* with P_GAPDH_. This knockout, when combined with the integration and overexpression of codon-optimized α1–2 mannosidase *MNS1*, may lethally alter the native glycosylation of the host’s glycoproteins, including structurally integral cell wall mannoproteins, and only cells that had rendered *MNS1* inactive through spontaneous mutations remained viable.

Among the amino acid substitution mutants, the majority of which were likely significantly deleterious (non-polar to polar or charged residues or vice versa), we identified one variant of *MNS1* with a methionine residue at position 260 mutated to isoleucine for further analysis. By comparing mannosidase homologs across different eukaryotic organisms, including *C. elegans*, *Drosophila melanogaster*, *Homo sapiens*, *Penicillium citrinum*, *Mus musculus*, and more (**Table S1**), we found that the methionine residue was not highly conserved across species, and leucine is the most frequent amino acid used in that position, followed by arginine and glycine (Fig. 1B). Given the structural similarity between leucine and isoleucine, we investigated this *MNS1* M260I mutant further because we posited that it may retain some degree of its enzymatic activity. We expressed K3 peptide in both GlcNAcMan5-and G0-glycoengineered *Δoch1* strains with the *MNS1* mutant and analyzed its glycan profile via intact protein LC-MS (Fig. 1C–D). The secreted peptide was predominantly decorated with the desired GlcNAcMan5 and G0 glycans, with some high-mannose glycan structures ranging from Man7 to Man11. The remaining presence of high mannose glycans could be attributed to incomplete cleavage of mannoses by Mns1p, and this decreased enzymatic activity supports the hypothesis that the M260I mutant allowed survival even when expressed under the control of the strong constitutive P_GAPDH_. Although the synthetic lethality between *Δoch1* and *MNS1* and this spontaneous *MNS1* mutant has not been previously reported, prior literature supports the observed difficulty in expressing this initiating enzyme of the glycosylation pathway. A truncated version of *MNS1* was used in a commercial Man5-glycoengineered strain (SuperMan5, generated using GlycoSwitch^®^ technology [11]), likely to improve strain fitness and ease for protein expression [14].

**Figure 2:**
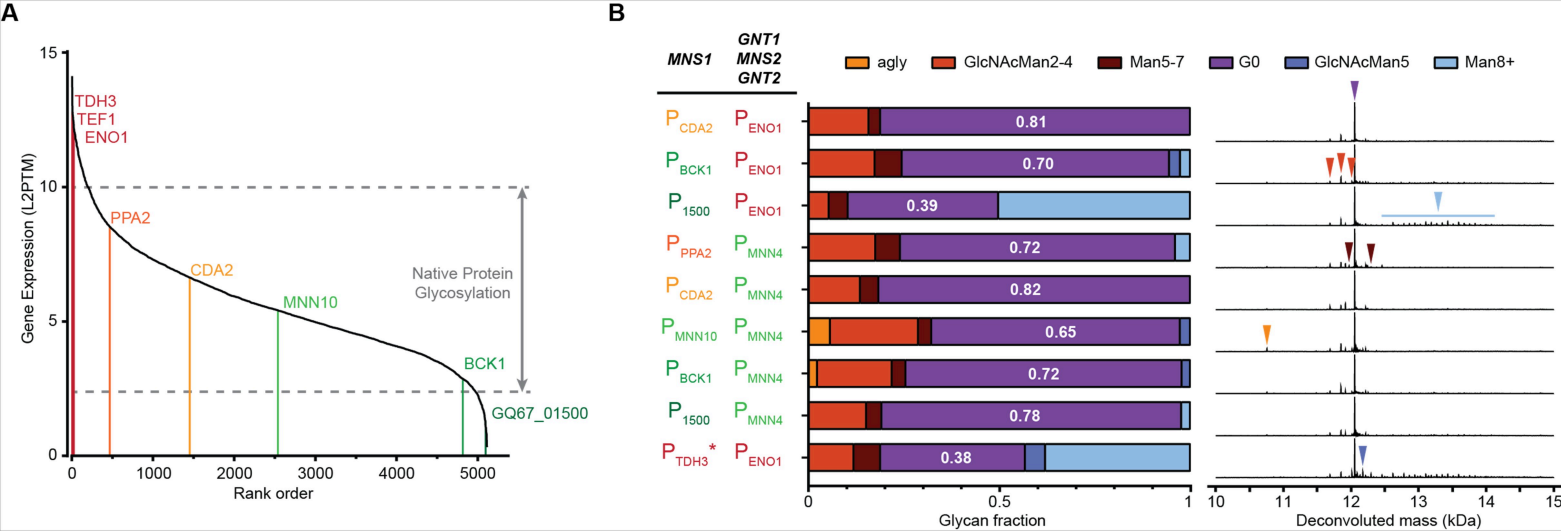
Attenuating wildtype *MNS1* expression enables G0 glycoengineering. A) Activity of native *K. phaffii* promoters for wildtype *MNS1* expression. B) Glycan profiles based on intact protein LC-MS of secreted K3 peptide from different G0 strains.

Because we observed cytotoxicity with strongly expressed *MNS1*, we next investigated whether viable transformants could be obtained by expressing wildtype *MNS1* with weaker promoters. We hypothesized that less active promoters could allow for similar level of overall mannosidase activity, but with the advantage of using less cellular resources. Indeed, *K. phaffii* genes like GAPDH and TEF1, whose promoters are commonly used for heterologous gene expression, are natively expressed tens to hundreds of times more than native glycosylation genes (**Figure S2**). To test if less active promoters could support the expression of wildtype *MNS1* without inducing cytotoxicity, we generated wildtype *MNS1* constructs expressed under the control of six additional promoters, selected based on their native gene expression – P_ENO1_, P_PPA2_, P_CDA2_, P_MNN10,_ P_BCK1_, and P_GQ67_01500_ (Fig. 2A). Four out of these six promoters have activities within the range of other genes in the glycosylation pathway of *K. phaffii*, and P_MNN10_, in particular, is a native glycosylation promoter. In addition to G0-glycoengineered strains with strong constitutive P_ENO1_-driven pathway expression, we also examined if other glycosidases and glycosyltransferases in the humanized pathway could be driven by the less active P_MNN4_ (**Table S2**). Wildtype *MNS1* expressed under strong *ENO1* promoter could not be integrated into either G0 strain, consistent with the observed synthetic lethality between *Δoch1* and strong constitutive expression of *MNS1*. G0 strains with *GNT1*, *MNS2*, and *GNT2* under the control of P_MNN4_ generally tolerated a higher expression level of wildtype *MNS1*, indicating a possible inverse connection between tolerable levels of *MNS1* expression and cellular stress from heterologous pathway overexpression.

We then cultivated these resulting G0 strains with successful *MNS1* integration and assayed the glycan profile of their secreted K3 peptide. All viable strains with the *MNS1* constructs produced homogeneous G0 glycans, and the presence of high-mannose structures was reduced in most G0 strains with wildtype *MNS1* compared to with mutant *MNS1*, despite the less active promoters used to drive expression (Fig. 2B).

Because glycoengineered strains have been reported to exhibit increased propensity to lysis [13] and growth defects [11], we characterized the cell wall of the glycoengineered strains against the wildtype strain. The wildtype strain was intense stained with Alcian blue, a polyvalent cationic dye that binds to negatively charged cell wall sugars [29], due to the phosphomannoses present on the mannan chain of cell-surface proteins (**Figure S3**). The disruption of OCH1 inhibits hypermannosylation, where most of the phosphomannoses reside, and thus significantly decreases Alcian blue staining. Integration of the mannosidase *MNS1* further precludes phosphomannose addition and decreases staining. After the subsequent introduction of *GNT1*, *MNS2*, and *GNT2*, however, the resulting glycoengineered strain showed significantly more Alcian blue staining compared to both *Δoch1* and *MNS1*-integrated strains. Because Alcian blue does not have high binding affinity to the terminal neutrally charged GlcNAc on G0 glycan, this increase in staining may result from the upregulation of native phosphomannosylation genes after extensive glycoengineering.

We also evaluated the resistance of different strains to Congo red and Calcofluor white. These two chemicals interfere with the construction and stress response of fungal cell wall, and their susceptibility can indicate the integrity of the cell wall [30]. We observed that *Δoch1* and *MNS1* integration greatly decreased the wildtype strain’s tolerance to Calcofluor white, but the integration of additional genes to G0 helped partially restore this phenotype (**Figure S3**). This observation indicated that disruption of OCH1 and integration of mannosidase significantly affect the integrity of the cell wall, but the addition of sugar moieties by *GNT1* and *GNT2* could help reinforce the cell wall. Assays with Congo red showed similar results: *Δoch1* and *MNS1* integrated strains showed zero growth, while G0-glycoengineered strains showed minimal growth.

We then examined the growth rate of the glycoengineered strains. At room temperature, the growth rate of glycoengineered strains was less than half of that for the wildtype strain, with the slowest one approximately 30% that of the wildtype (**Figure S4**). Furthermore, six of nine glycoengineered strains showed a significant decrease in growth rates upon *MNS1* knock-in, though there was no apparent correlation between the decrease in growth rate and the activity of *MNS1* promoter. Additionally, when comparing strains with the same *MNS1* constructs but differentially expressed *GNT1*, *MNS2*, and *GNT2* (using either ^P^_ENO1_ or ^P^_MNN4_), the reduction in metabolic burden by using less active promoters for heterologous pathway expression was not reflected in any significant differences in growth rate.

To better understand which engineering changes predominantly impacted growth, we performed additional growth rate assays (**Figure S5**). We observed that the integration of heterologous pathway genes resulted in more than 40% decrease in growth rate in wildtype *K. phaffii* with intact *OCH1*. In the *Δoch1* strain, however, subsequent pathway integration had no negative impact on growth, and G0-engineered strains had higher growth rates than the *Δoch1* strain. This partial recovery of growth defects contradicts previous reports of serious growth defects after integration of *MNS2* and *GNT2* [11] and may result from using less active promoters for the heterologous genes expressed. We also examined secretion of the K3 peptide from select glycoengineered strains and found different *MNS1* constructs or expression levels of other heterologous pathway enzymes did not seem to greatly impact K3 peptide secretion (**Figure S6**).

**Figure 3:**
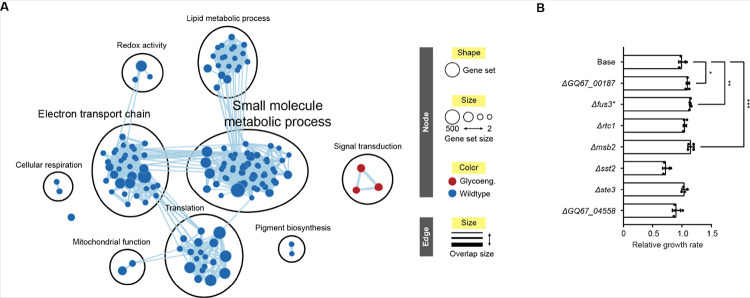
Improving growth of a glycoengineered strain via transcriptomics-informed gene knockout. A) Pathway gene set enrichment map of wildtype vs. a glycoengineered strain. B) Restoration of growth rate defects of the glycoengineered strain through gene knockouts in the MAPK cascade signal transduction pathway.

Cell growth is a complex phenotype, and attempts for its improvement in glycoengineered strains have largely relied on random mutagenesis [13], [31]. While effective, random mutagenesis can be time- and labor-intensive, and the engineering insights we could gain from such genome-scale perturbations are often limited. RNA sequencing has the potential to guide genetic perturbations in a more targeted manner than this approach. We sequenced the transcriptomes of several G0-glycoengineered strains and compared them to that of wildtype *K. phaffii*, and performed pathway enrichment analysis to assess variations in transcriptomic states. We observed that, in the wildtype strain, pathways related to electron transport, metabolic processes, and redox activity were upregulated, while those related to signal transduction were upregulated in the G0 glycoengineered strain (Fig. 3A). This difference would explain the observed growth defects since the wildtype strain exhibits high metabolic activity, while signal transduction (the yeast mitogen-activated protein kinase (MAPK) cascade) mediates cell growth and stress response [32]. Based on this finding, we targeted select heavily upregulated but relatively non-essential genes in the MAPK pathway for knockout to examine if such genetic perturbations could partially restore cell growth (**Table S3**). Three of seven genomic perturbations had positive impact on cell growth, with *Δmsb2* yielding a 15% increase in growth rate (Fig. 3B), thus demonstrating that transcriptomics datasets can inform engineering targets to more effectively improve undesired phenotypes like growth defects.

## Discussion

In this study, we have demonstrated a host-informed approach to engineer the glycosylation pathway of *K. phaffii* to yield strains capable of producing homogeneous N-linked G0 glycans. During the construction of this heterologous pathway, we identified a previously unreported synthetic lethality between *Δoch1* and *MNS1*, evident by spontaneous, unlinked mutations in the open reading frame of the heterologous gene after disrupting *OCH1*. Truncated variants of *MNS1* have been previously used for constructing glycoengineered strains with increased viability and stability [14]. Furthermore, some of the variations in enzymatic efficiency observed in combinatorial library screens could possibly be explained by mutations in the gene constructs after cassette integration, in addition to the differences in catalytic turnover rate of heterologous mannosidases and the effectiveness of subcellular localization sequences [10]. Confirmation by Sanger sequencing of each individual clone within those combinatorial libraries is resource-intensive and was very likely cost prohibitive in the 2000s.

We isolated a spontaneously occurring *MNS1* mutant that enabled viable engineering of the G0 pathway due to its reduced enzymatic activity. Similar reduction in *MNS1* activity was then achieved by using promoters (P_CDA2_, P_MNN10_, and P_BCK1_) less active than the GAPDH promoter. The majority of *MNS1* constructs tested also generated more homogeneous glycosylation profiles on secreted K3 peptide, compared to the *MNS1* mutant, despite the reduction in promoter strength. Tuning gene expression to appropriate host-informed levels proved effective in heterologous pathway engineering here and, in this case, circumvented the synthetic lethality while improving pathway processivity by using the more catalytically active native *MNS1* sequence. Consistent with prior reports, we observed significant growth defects in glycoengineered strains, and gene set enrichment analysis of transcriptomic data supported the observation as wildtype strain was more metabolically active, while the glycoengineered strains had upregulated MAPK cascade. This analysis also guided gene knockouts to partially rescue growth defects, with *Δmsb2* yielding a 15% increase in cellular growth. Contrary to random mutagenesis techniques, transcriptomics-informed genetic engineering allowed us to alleviate this undesired phenotype in a direct, more targeted manner. Interestingly, one prior report partially restored growth in glycoengineered *S. cerevisiae* by using transposon-based *in vivo* mutagenesis to upregulate *BEM4*, another regulatory gene in the MAPK cascade [31]. This signaling transduction pathway could thus be a good modulation target for growth regulation, although the exact genetic perturbation needed likely differs by strain and existing engineering.

In addition to K3 peptide, we have expressed additional glycoproteins, including a protein subunit vaccine candidate [33] and a monoclonal antibody, in the generated glycoengineered strains, but high mannose structures persist as measured by intact MS analysis, especially for the monoclonal antibody (**Figure S7**). Glycosylation pathway is inextricably linked with many other protein folding and quality control pathways, most notably endoplasmic reticulum-associated degradation (ERAD) [34]. A number of Golgi-resident mannosyltransferases can also compete with *GNT1* and *GNT2* for the same substrate [35]. These results indicate that to properly glycosylate these more complex, pharmaceutically relevant proteins, additional engineering to remodel the secretory pathway is likely required – introduction of protein folding chaperones and knockout of promiscuous mannosyltransferases could be beneficial to increase glycan homogeneity on more complex glycoproteins [36]–[38].

Construction of a suitable alternative host for the expression of therapeutic glycoproteins is important in increasing the accessibility of certain biopharmaceuticals and could accelerate their development. In this study, we provide evidence that host-informed approaches can improve glycoengineering. The tuning of the N-linked glycosylation pathway employed here could be applied engineering of other multigene pathways as well. As interest grows in developing *K. phaffii* as a host for more advanced synthetic biology and metabolic engineering, transcriptomics and other -omics-based approaches may aid in further refining the engineering of these complex pathways to enable improved strains producing heterologous glycoproteins.

## Supplementary Material

Supplementary Files

This is a list of supplementary files associated with this preprint. Click to download.

- SupplementalInformation.zip

## Data Availability

The data that support the findings of this study are available in the supplementary material of this article or from the corresponding author upon request.
